# Effect of *Astragalus sinicus* Ethanol Extract on Menopausal Symptoms in an Ovariectomized (OVX) Mouse Model

**DOI:** 10.4014/jmb.2510.10033

**Published:** 2026-01-23

**Authors:** Holim Jin, Yongjin Lee, Yoon-A Shin, YongMin Lim, Myung Rye Park, Youn Kyu Kim, Cheol Moon, Young-Jin Son

**Affiliations:** 1 Department of Pharmacy, Sunchon National University, Suncheon 57922, Republic of Korea; 2 Department of Nutritional Science & Food Management, Ewha Womans University, Seoul 03760, Republic of Korea; 3 Korea Research Institute Bio Science Co., Ltd., Yeoju 12668, Republic of Korea

**Keywords:** Menopause, *Astragalus sinicus*, Ovariectomized, Bone mineral density, MCF-7

## Abstract

As the aging population grows, the proportion of postmenopausal women is also increasing. Menopause can cause various symptoms and reduce quality of life. Hormone replacement therapy (HRT) is a common treatment to alleviate these symptoms, but long-term use carries the risk of side effects such as breast cancer and endometriosis, highlighting the need for new alternatives. *Astragalus sinicus* is a plant native to East Asia, primarily used to improve rice cultivation in paddies. However, research on its potential as a medicinal resource remains limited. Therefore, this study aimed to evaluate the potential of *A. sinicus* ethanol extract as a treatment for menopause-related symptoms using the MCF-7 breast cancer cell line and ovariectomized (OVX) mice. *In vitro* experiments confirmed that *A. sinicus* ethanol extract improved the mRNA expression of menopause-related genes and exhibited estrogenic activity. Additionally, *in vivo* experiments demonstrated that *A. sinicus* ethanol extract effectively alleviated menopausal symptoms. These findings suggest that *A. sinicus* ethanol extract may serve as a novel alternative for addressing menopause-related health issues.

## Introduction

As the world enters an aging society, the proportion of elderly women in the total population is increasing [1]. According to the UN World Population Prospects 2024 report, by 2030, women will make up 54% of the 2.3 billion people aged 30 and over worldwide, and they are expected to account for 60% of the population aged 80 and over [2, 3]. This indicates that the number of postmenopausal women is increasing globally, as most women experience menopause between the ages of 45 and 55 [4].

Menopause refers to the phenomenon in which estrogen secretion decreases due to ovarian aging, leading to the complete cessation of ovulation and menstruation. In the early stages of menopause, the number of ovarian granulosa cells decreases, resulting in a reduction in the production of estradiol and inhibin, followed by an increase in follicle-stimulating hormone (FSH) and luteinizing hormone (LH) levels. This process, combined with the decline in estrogen levels, disrupts the hypothalamic-pituitary-ovarian axis, causing menstrual cycles to become irregular until they completely stop [5-9]. Although menopause is a natural process in a woman's life, the symptoms and related diseases can interfere with daily life and reduce quality of life. Menopausal symptoms include vasomotor symptoms, urogenital symptoms, psychological symptoms, as well as conditions such as hypertension, weight gain, and breast size reduction. Vasomotor symptoms affect approximately 75% of menopausal women and include hot flashes, night sweats, palpitations, and migraines. About 60% of women experience urogenital symptoms, such as vaginal atrophy, urethral atrophy, and sexual dysfunction. Over time, postmenopausal complications such as cardiovascular disease and osteoporosis may develop [10-14]. Hormone replacement therapy (HRT) is widely used to treat menopause; however, it can increase the risk of side effects such as breast cancer and stroke [15]. Consequently, phytoestrogens, plant compounds found in natural foods such as soybeans, flaxseeds, and whole grains, are emerging as safer alternatives [16-18].

Estrogen is an important steroid hormone secreted by the ovaries in the female body. It exists in three forms: estrone (E1), 17β-estradiol (E2), and estriol (E3). Estrogen plays a crucial role in reproductive development, menstrual cycle regulation, bone health maintenance, and cardiovascular protection [19]. When estrogen binds to the estrogen receptor (ER), it exerts various physiological effects through gene expression regulation and cell signaling pathways. Specifically, ER dimerizes and then binds to the estrogen response element (ERE) to form the ER-ERE complex. This complex regulates the expression of estrogen-responsive target genes such as the progesterone receptor (PR) and presenilin-2 (pS2), thereby stimulating cell proliferation and differentiation [20-22].

Phytoestrogens are plant-derived compounds that are structurally and functionally similar to 17β-estradiol, or they exhibit estrogenic activity. They have a diphenolic structure, and several types have been identified, including lignans, isoflavonoids, and stilbenes [23-26]. Among these, isoflavonoids have stronger estrogenic activity than other phytoestrogens. In particular, they have an affinity for estrogen receptor beta (ERβ) that is approximately five times higher than for the alpha receptor (ERα) and exhibit estrogenic activity about 10,000 times lower than that of estradiol. Therefore, they are considered relatively safe but less effective than hormone replacement therapy (HRT). In addition to their selective estrogenic effects, they can also contribute to reducing free radicals through a tyrosine kinase inhibition mechanism [26-28]. However, an important point is that isoflavones and E2 exhibit the same level of biological activity when applied at sufficiently high concentrations to elicit a maximal response. This may indicate that the estrogen receptor complex formed by E2 and isoflavonoids functions equivalently. Previous studies have shown that phytoestrogens exhibit various biological activities, including anticancer, antioxidant, antiproliferative, and antiangiogenic properties [26, 28].

*Astragalus sinicus* is a perennial herbaceous plant native to East Asia, especially Japan, southern China, and central Korea, and belongs to the Fabaceae. This plant can fix nitrogen through symbiosis with soil bacteria during its growth, playing an important role in soil fertility. Due to these characteristics, it is widely known for its use in rice fields to enhance rice cultivation techniques. *A. sinicus* is considered safe, with soft young leaves and stems, and has been traditionally used as a folk remedy for treating phlegm, sore throat, external bleeding, and shingles. Additionally, its flowers are brewed and consumed as herbal tea. However, most research on *A. sinicus* has focused on its role as a natural fertilizer for enhancing rice cultivation, while studies on its potential as a medicinal plant remain relatively limited [29,30].

The acetone extract of *A. sinicus* seeds was found to be rich in total flavonoids and total phenols, and it exhibited antioxidant effects, as measured by DPPH radical scavenging activity [31]. Additionally, a study analyzing the antioxidant and anti-inflammatory effects of methanol extracts of *A. sinicus* in HaCaT cells reported significant results. Specifically, the *A. sinicus* methanol extract demonstrated anti-inflammatory effects by regulating the NF-κB inflammatory signaling pathway and reducing the production of inflammatory cytokines such as IL-1, IL-6, IL-8, and TNF-α [32]. Furthermore, the *A. sinicus* methanol extract exhibited estrogenic activity in an experiment using MCF-7 cells and a luciferase reporter gene [33]. Additionally, *A. sinicus* ethanol (ASE) extract has been reported to alleviate the aging response of dermal cells and provide a protective effect against photoaging caused by UV exposure [30]. According to previous studies, the whole plant of *A. sinicus* contains trigonelline, canavanine, choline, soyasaponin, and sapogenol, while its seeds contain canavanine, canaline, homoserine, coumestrol, and daidzin [34].

While previous studies have employed various organic solvents such as methanol and acetone to extract this material, this study selected ethanol as the extraction solvent. This decision was based on two primary advantages of ethanol regarding the development of nutraceuticals and pharmaceuticals. First, ethanol is classified as Generally Recognized As Safe (GRAS) by regulatory agencies, including the U.S. FDA, for food and pharmaceutical manufacturing. This provides a significant safety advantage when preparing extracts intended for human consumption [35]. In contrast, solvents like methanol and acetone pose risks of potential toxic residues, necessitating additional purification processes that can hinder their application as functional food ingredients [36]. Second, ethanol possesses a broad polarity range, enabling the efficient extraction of both polar and non-polar compounds [37]. This property is particularly advantageous for recovering a wide spectrum of bioactive constituents, such as polyphenols and phytoestrogens. Furthermore, aqueous ethanol systems have been reported to maximize both the extraction yield and antioxidant potential of phenolic compounds [38].

Therefore, this study aimed to evaluate the potential of a safe ethanol extract of *A. sinicus* as a functional material for alleviating menopausal symptoms. Specifically, we investigated its estrogen-like activity *in vitro* and evaluated its therapeutic effects on key postmenopausal changes, such as obesity, uterine atrophy, dyslipidemia, and osteoporosis, in an ovariectomized mouse model.

## Materials and Methods

### Preparation of ASE Extract

*Astragalus sinicus* was collected in early May from the farmland Hampyeong-gun, Jeollanam-do. It was naturally dried under sunlight for 2 days, two kg of dried whole plant (roots, stems, leaves, and flowers) of *A. sinicus* were then extracted with 40 L of 70% ethanol/water mixture (*v/v*) at 80°C for 6 h. The extract was filtered with filter cloth, and the filtrate was concentrated using a vacuum evaporator under the following conditions: 10~20 kPa, 40 rpm, and 50°C. Subsequently, it was freeze-dried for 48 h, yielding 0.5 kg of ASE powder. The freeze-dried powder was vacuum-sealed and stored frozen for experimental use [30].

### Cell Culture

For this study, the human breast cancer cell line MCF-7 was purchased from the Korean Cell Line Bank. MCF-7 cells were cultured in DMEM (Dulbecco's Modified Eagle's Medium; Republic of Korea) containing 100 U/ml P/S (penicillin/streptomycin) and 10% FBS (fetal bovine serum; Invitrogen Life Technologies, USA). All cells were cultured in an incubator at 37°C under 5% CO_2_.

### Design of Animal Experiments

All animal experiments were performed in accordance with the guidelines of the Animal Experiment Ethics Committee of Sunchon National University (SCNU IAUC-2024-14). For this animal experiment, four-week-old female ICR mice were purchased from GBIO (Republic of Korea). After a seven-day acclimation period, the mice were housed in an animal room maintained at 22 ± 3°C with a 12-h light/dark cycle. They had free access to commercially available food and water. Subsequently, five-week-old ICR female mice were randomly assigned to six groups (eight mice per group): a control group (Sham), OVX (ovariectomized), OVX + positive control (estradiol, 0.5 mg/kg BW), OVX + low-concentration ASE extract (30 mg/kg BW), OVX + medium-concentration ASE extract (100 mg/kg BW), and OVX + high-concentration ASE extract (300 mg/kg BW) (Fig. 3A).

An incision was made on the backs of the mice in the OVX, OVX + Estradiol, OVX + low-concentration ASE extract, OVX + medium-concentration ASE extract, and OVX + high-concentration ASE extract groups, and their ovaries were removed. In the sham group, the same incision was made without removing the ovaries. Estradiol was injected subcutaneously three times a week, and ASE extract was administered orally once a day for 6 weeks. Food intake was measured three times a week, whereas body weight was recorded once a week. After 6 weeks, the mice were sacrificed, and the uterus, Parametrial white adipose tissue (pWAT), Abdominal adipose (AA), kidneys, and liver were weighed. Blood samples were collected and centrifuged at 5,000 rpm for 5 min; the serum was then immediately stored at -80°C. The femurs were fixed in 10% formalin and subsequently stored in 70% ethanol.

### Analysis of ASE Extract Cytotoxicity

Cytotoxicity was analyzed using a Cell Counting Kit-8 (CCK-8; Tomado Molecular Technology, Japan). Specifically, MCF-7 cells were seeded at 6.7 × 10^3^ cells per well in a 96-well culture plate. The next day, the cells were treated with ASE extract at concentrations of 10, 20, and 50 μg/ml, and estradiol (100 μM) was used as a positive control. After 48 h of incubation, the supernatant was removed, and 100 μl of 10% CCK-8 buffer was added to each well. The plates were then incubated for 2 h at 37°C, after which the absorbance was measured at 450 nm using a microplate reader (Bionice, Republic of Korea).

### Real-Time Qantitative Reverse Transcription PCR (RT-qPCR) Aalysis of ASE Extract

MCF-7 cells were seeded in 6-well culture plates at a density of 3 × 10^5^ cells per well. The following day, the medium was replaced with 2.5% charcoal-stripped DMEM containing the sample, and cells were harvested after 48 h. Primers were designed using the Primer3 design program (Table 1). Total RNA was isolated from both cells and uterine tissue using TRIzol reagent (Thermo Fisher Scientific Inc., USA). cDNA was synthesized using an M-MLV cDNA synthesis kit (Enzynomics, Republic of Korea). Real-time qPCR was performed using a Real-Time PCR detection system (Bio-Rad) with TOPreal qPCR 2× PreMIX (Bio-Rad, USA). The mRNA levels of the target genes were determined by the 2^^−ΔΔCT^ method, with glyceraldehyde-3-phosphate dehydrogenase (GAPDH) used as the internal control.

### Western Blot Analysis of ASE Extract

Proteins were extracted using a lysis buffer containing aprotinin (10 μg/ml), DTT (1 mM), EDTA (2 mM), leupeptin (5 μg/ml), NaCl (150 mM), 1% (v/v) Igepal CA-630, sodium orthovanadate (1 mM), pepstatin (2 μg/ml), sodium fluoride (1 mM), and Tris-HCl (20 mM, pH 7.5). A total of 20 μg of extracted protein was separated on a 10% SDS-PAGE gel by electrophoresis, and the proteins were transferred to polyvinylidene difluoride (PVDF) membranes (Amersham Biosciences, USA). After incubation with primary antibodies (p-ERK, t-ERK, p-AKT, t-AKT) at 4°C for 24 h, the membranes were washed with TBS containing 1% Tween 20. Next, they were incubated with a horseradish peroxidase (HRP)-conjugated secondary antibody at room temperature for 1 h, followed by additional washes with TBS containing 1% Tween 20. Protein detection was performed using the SuperSignal West Pico Chemiluminescent Substrate (Pierce Chemical, USA), and the intensity of the protein bands was analyzed using the MicroChemi 4.2 system (DNR Bio-imaging System, USA).

### Serum Analysis

Serum was used to analyze blood lipid levels (triglycerides, total cholesterol, high-density lipoprotein (HDL)-cholesterol, low-density lipoprotein (LDL)-cholesterol), as well as alkaline phosphatase and calcium, which are associated with cardiovascular health. These biomarkers were measured using a dry-type clinical chemistry automatic analyzer (Fuji Drichem 4000i).

### Micro-Computed Tomography Analysis

Femurs were scanned and analyzed using a high-resolution micro-computed tomography (CT) scanner (SkyScan 1173, Skyscan NV, Kontich, Belgium) and SkyScan Data Viewer software (Skyscan NV).

### Statistical Analysis

All data are expressed as mean ± SEM. statistical analyses were performed using the SPSS v21.0 (IBM, Armonk, USA). One-way analysis of variance (ANOVA) followed by Tukey’s post-hoc test were used to analyze all parameters between groups. *P*-values < 0.05 were considered statistically significant, and representative images are displayed.

## Results

### Confirmation of Cytotoxic Effects of ASE Extract

**Fig. 1:**
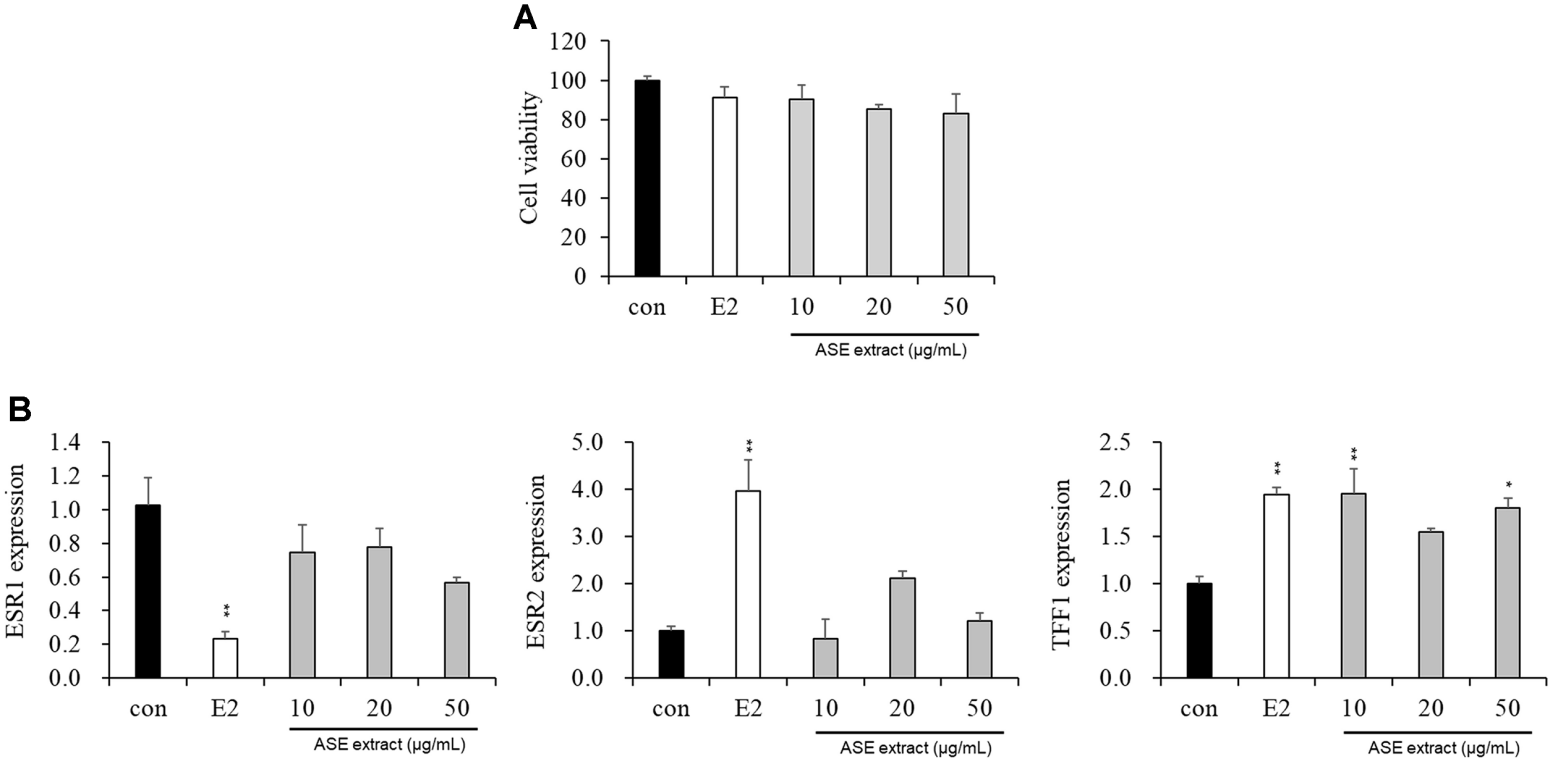
Analysis of suppression effects using various genes related to female menopause. (**A**) Cells were treated with the indicated concentrations of ASE extract for 48 h and viability was determined by CCK-8 assay. (**B**) Effect of ASE extract on estrogen receptor-mediated *ESR1*, *ESR2*, and *TFF1* expression for 48 h. Glyceraldehyde-3-phosphate dehydrogenase (GAPDH) was used as housekeeping, and E2 was used as a positive control. Data are presented as means ± SEM values of three independent replicates. Data were analyzed using one-way ANOVA followed by Tukey’s post-hoc test. versus the control group; * *p* < 0.05, ** *p* < 0.01.

To determine the cytotoxicity of the ASE extract, MCF-7 cells, a breast cancer cell line, were treated with ASE extract (10, 20, and 50 μg/ml) or the positive control E2 (100 μM). Cytotoxicity was then measured 48 h later using Cell Counting Kit-8. As a result, no significant cytotoxicity was observed in the ASE extract (10, 20, and 50 μg/ml) and positive control E2 (100 μM) treatment groups (Fig. 1A).

### Effect of ASE Extract on the mRNA Expression of Genes associated with Menopause

To analyze the effect of ASE extract on the menopause mechanism, MCF-7 cells were treated with ASE extract (10, 20, and 50 μg/ml) or the positive control E2 (100 μM) and cultured for 48 h. The cells were then harvested, total mRNA was extracted, cDNA was synthesized, and real-time qPCR was performed to analyze the transcriptional expression of menopause-related genes, including *ESR1* (ERα mRNA), *ESR2* (ERβ mRNA), and *TFF1* (pS2 mRNA). As a result, the ASE extract decreased the mRNA expression of *ESR1*, similar to the positive control E2, whereas the mRNA expression of *ESR2*, and *TFF1* increased (Fig. 1B).

### Effect of ASE Extract on Menopause-Related Changes in Signaling Proteins

**Fig. 2:**
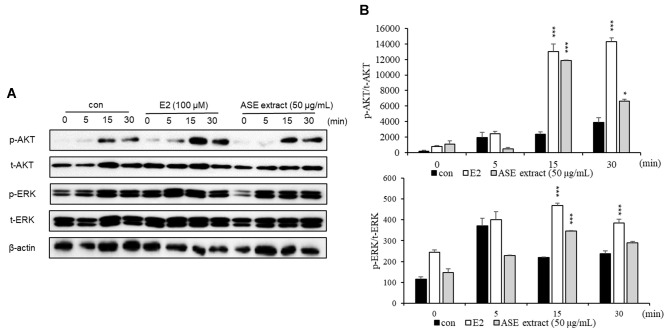
Changes in expression of signaling proteins related to female menopause by ASE extract. (**A**) After 0, 5, 15, and 30 min of treatment with ASE extract, p-AKT, t-AKT, p-ERK, and t-ERK were measured by Western blotting. (**B**) Corrected based on the amount of β-actin loading. E2 was used as a positive control. Data are presented as means ± SEM values of three independent replicates. Data were analyzed using one-way ANOVA followed by Tukey’s post-hoc test. versus the control group; *p < 0.05, ***p < 0.001.

To analyze the effect of ASE extract on estrogen signaling after menopause, MCF-7 cells were divided into three groups: a control group (not treated with ASE or E2), a positive control group (treated with E2 at 100 μM), and an experimental group (treated with ASE extract at 50 μg/ml). The cells were harvested at 0, 5, 15, and 30 min, followed by protein extraction, quantification, and Western blot analysis to examine changes in total protein and phosphorylation levels of AKT and ERK, key indicators of estrogen signaling. As a result, both the positive control group (E2-treated) and the ASE-treated group showed increased estrogen activity compared to the control group (Fig. 2). This suggests that ASE treatment enhances estrogen signaling.

### Effects of ASE Extract on Food Intake and Changes in Body Weight in Ovariectomized Mice

**Fig. 3:**
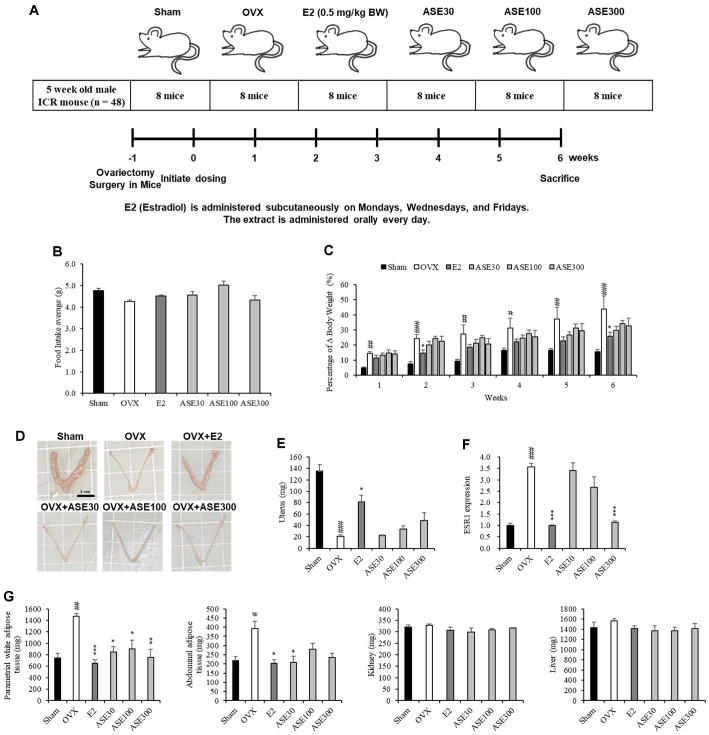
Effects of ASE extract on Morphological Changes in Ovariectomized Mice. (**A**) Experimental design and experimental plan for ASE Extract in OVX-induced mice. (**B**) Average food intake over 6 weeks. (**C**) Weight gain percentage over 6 weeks. (**D**) Representative image of the uterus for each group. (**E**) Comparison of Uterine Weight. (**F**) Changes in *ESR1* mRNA expression in the uterus. Glyceraldehyde-3-phosphate dehydrogenase (GAPDH) was used as the internal control. (**G**) pWAT, AA, Kidney, and Liver Weight Graph. Data are presented as mean ± SEM values (*n* = 8). Data were analyzed using one-way ANOVA followed by Tukey’s post-hoc test. ^#^*p* < 0.05, ^##^*p* < 0.01, ^###^*p* < 0.001 compared to the sham group; *p < 0.05, **p < 0.01, ***p < 0.001 compared to the non-treated OVX group.

To analyze the effect of ASE extract on body weight in ovariectomized (OVX) mice, daily food intake and weekly body weight were measured for six weeks, and the rate of change was calculated. The experimental results showed that there was no significant difference in food intake among the groups (Fig. 3B). However, body weight significantly increased in the OVX group compared to the Sham group, whereas it significantly decreased in the E2, ASE30, ASE100, and ASE300 groups compared to the OVX group (Fig. 3C). These findings suggest that ASE extract intake has a suppressive effect on body weight gain.

### Effects of ASE Extract on the Uterus of Ovariectomized Mice

Since the uterus is regulated by estrogen concentration, this study analyzed the effect of ASE extract on the uterus in mice that were induced into menopause by OVX. To achieve this, uterine weight, uterine images, and changes in the mRNA expression of *ESR1* in the uterus were examined. As a result, the uterine shape and weight were reduced in the OVX group compared to the Sham group. However, uterine weight increased in a dose-dependent manner with ASE extract concentration, and the mRNA expression of *ESR1* in the uterus decreased similarly to that in the positive control group, E2 (Fig. 3D-3F).

### Effects of ASE Extract on Tissue Weight Changes in Ovariectomized Mice

To analyze the effect of ASE extract on tissue weight in mice with OVX-induced menopause, the tissue weights of the liver, kidney, AA, and pWAT were measured. The results showed no significant differences between the groups in liver and kidney weights. However, in adipose tissues such as AA and pWAT, a tendency toward suppressed fat accumulation was observed in the E2, ASE30, ASE100, and ASE300 groups (Fig. 3G).

### Effects of ASE Extract on Blood ALP and Ca Level Changes in Ovariectomized Mice

**Fig. 4:**
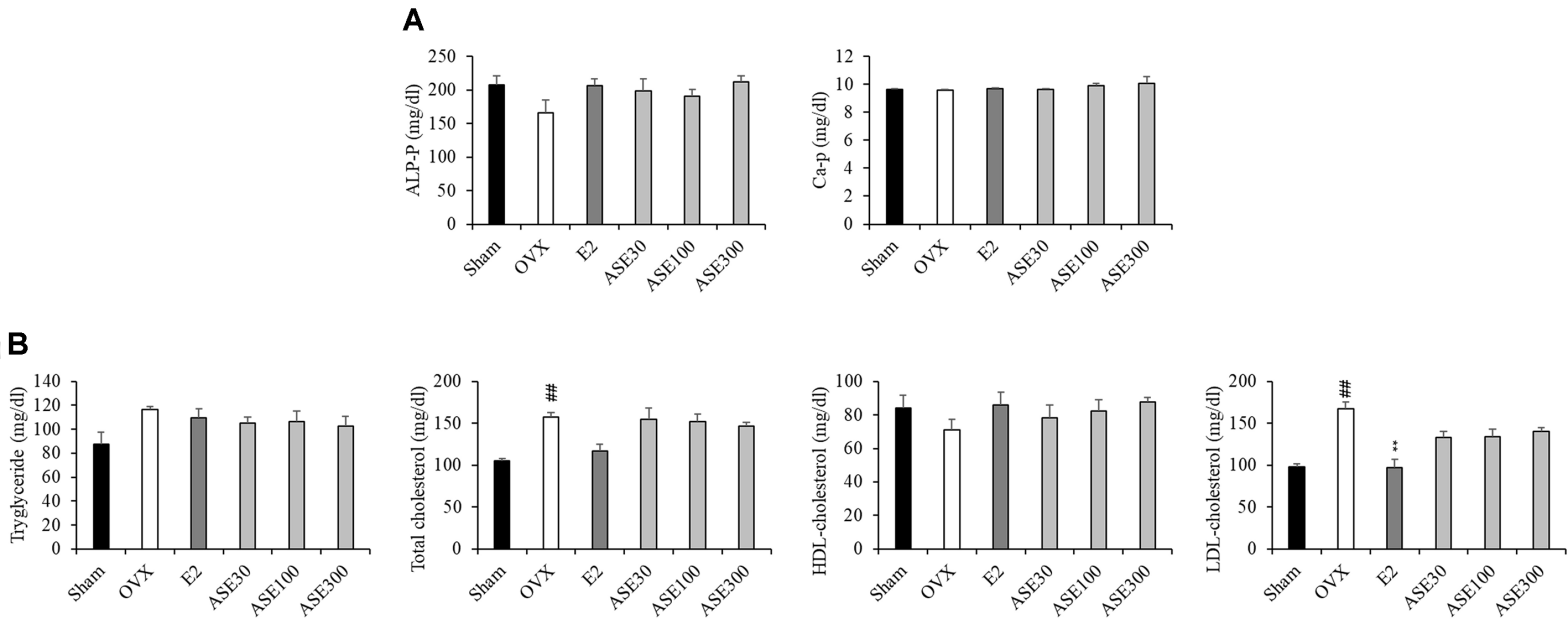
Effects of ASE extract on Bone Metabolism and Cardiovascular Markers in Ovariectomized Mice. (**A**) Serum levels of ALP, Ca marker were detected using kits. (**B**) Serum levels of cardiovascular marker were detected using kits. Data are presented as mean ± SEM values (*n* = 8). Data were analyzed using one-way ANOVA followed by Tukey’s post-hoc test. ^##^*p* < 0.01 compared to the sham group; **p < 0.01 compared to the non-treated OVX group.

Osteoblasts produce alkaline phosphatase, which hydrolyzes phosphate and promotes bone mineralization. Additionally, osteoblasts regulate the pH of the bone through alkalinization and induce calcium deposition by controlling the transport of phosphate and calcium ions. This process neutralizes acids that may be generated during mineral deposition and ensures the stable formation of bone tissue. Therefore, in this study, to analyze the effects of ASE extract on blood ALP and Ca levels in mice with OVX-induced menopause, the amounts of alkaline phosphatase and Ca in mouse serum were measured. As a result of the experiment, alkaline phosphatase levels tended to increase in response to ASE extract, but no significant change was observed in calcium levels (Fig. 4A).

### Effects of ASE Extract on Changes in Cardiovascular Markers in Ovariectomized Mice

To analyze the effects of ASE extract on blood lipid levels related to cardiovascular health in mice with OVX-induced menopause, the levels of triglycerides (TG), total cholesterol (TC), HDL-cholesterol (HDL-c), and LDL-cholesterol (LDL-c) in mouse serum were measured. As a result of the experiment, TG, TC, and LDL-c levels increased in the OVX group but decreased in the ASE extract group. On the other hand, HDL-c levels decreased in the OVX group but increased in the ASE extract group (Fig. 4B).

### Effects of ASE Extract on Bone Loss in Ovariectomized Mice

**Fig. 5:**
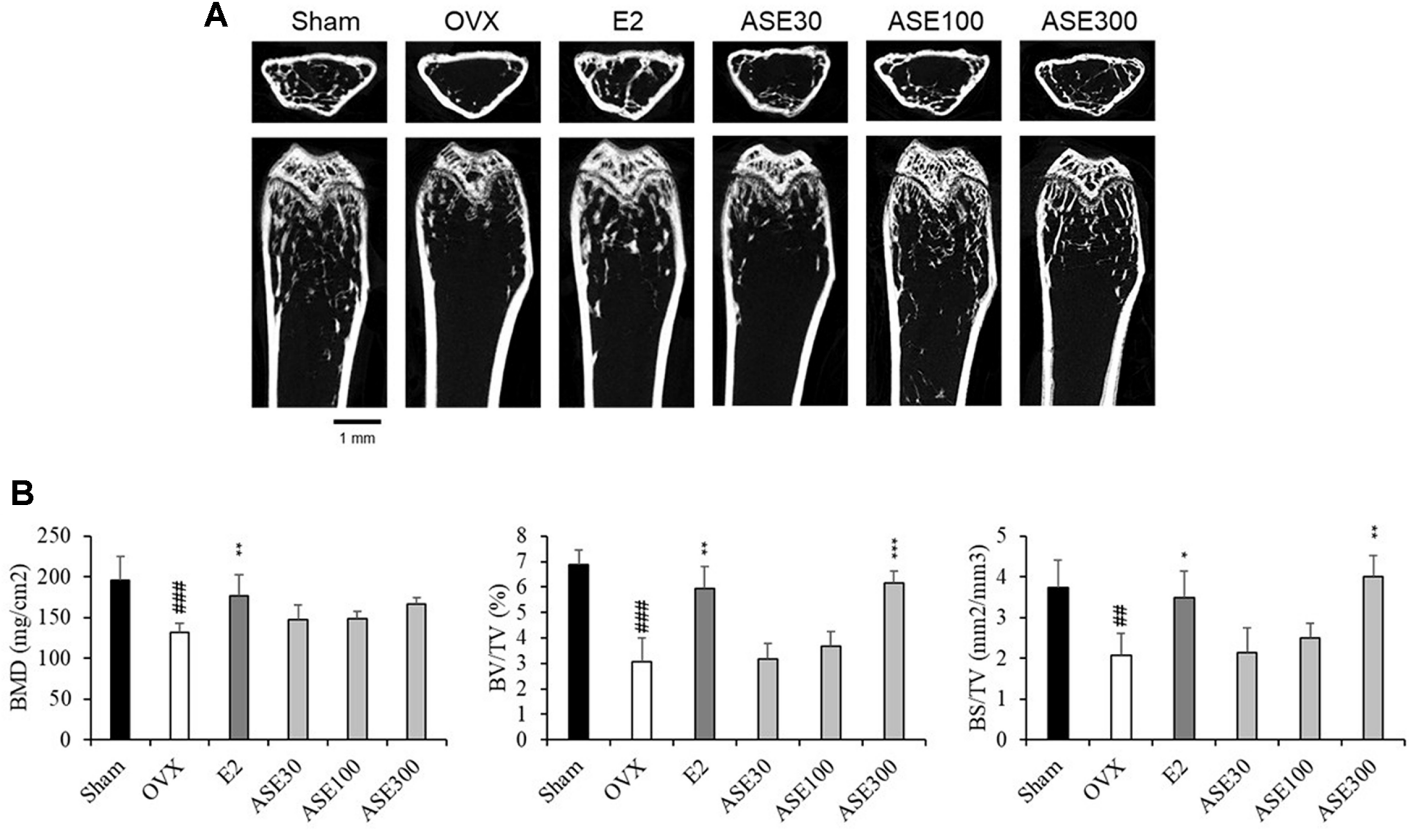
Effects of ASE extract on Bone Loss in Ovariectomized Mice. (**A**) Representative micro-CT images. (**B**) Quantification of microstructural parameters, including BMD, BV/TV, and BS/TV. Scale bar, 1 mm. Data are presented as mean ± SEM values (*n* = 8). Data were analyzed using one-way ANOVA followed by Tukey’s post-hoc test. ^##^*p* < 0.01, ^###^*p* < 0.001 compared to the sham group; *p < 0.05, **p < 0.01, ***p < 0.001 compared to the non-treated OVX group.

To analyze the effect of ASE extract on bone health in OVX-induced menopausal mice, bone mineral density (BMD), bone volume fraction (BV/TV), and bone surface-to-volume ratio (BS/TV) were measured by scanning the femurs of the mice using micro-CT. The results showed that BMD, BV/TV, and BS/TV were significantly reduced in the OVX group compared to the Sham group. However, administration of ASE extract at three doses (30, 100, and 300 mg/kg) for six weeks tended to improve these bone structural indices in a dose-dependent manner in OVX mice. Notably, the 300 mg/kg ASE extract exhibited a similar effect to estradiol (E2), which was used as a positive control in this study (Fig. 5). These results suggest that ASE may contribute to the inhibition of OVX-induced osteoporosis in a dose-dependent manner.

## Discussion

As the global population ages, the proportion of postmenopausal women is increasing. With the average life expectancy rising to 70 years, many women will spend more than one-third of their lives in a menopausal state. Consequently, the health of postmenopausal women has become a major global concern [13, 39]. Hormone replacement therapy (HRT) has traditionally been used to treat menopause and is known to be effective in alleviating menopausal symptoms. However, it is associated with various side effects, including an increased risk of breast cancer and stroke [40-43]. Natural plant-derived substances have long been used to treat various diseases, and their long-term use has rarely been linked to adverse side effects [44, 45]. As a result, alternative therapies for alleviating menopausal symptoms are gaining considerable attention. Phytoestrogens found in soybeans are one such alternative therapy. These compounds function similarly to estrogen by binding to estrogen receptors and mimicking their action, thereby helping to alleviate certain menopausal symptoms [46-48]. Soy-based products and the isoflavonoids they contain are increasingly recognized as safer and more effective alternatives for addressing health issues associated with hormonal imbalances [48, 49].

*A. sinicus* is the largest genus in the Fabaceae family, belonging to the subfamily Papilionoideae and the tribe Astragaleae. It is mainly distributed in Europe, Asia, and North America but can also be found in the mountainous regions of Africa and South America. Additionally, it is widespread in temperate regions worldwide [50]. The chemical composition of *A. sinicus* includes various compounds such as triterpenoid saponins, flavonoids, and glycosides [51]. Previous studies have reported its positive estrogenic activity [13].

MCF-7 cells are widely used in breast cancer research and serve as a major model for analyzing tumor biology and the mechanisms of hormone action [52]. In particular, they are commonly used to evaluate the estrogenic effects of phytoestrogens due to their stable estrogen sensitivity and high reproducibility [53]. This vertebrate-derived cell line harbors estrogen receptors (ERs), primarily ERα and ERβ. ERα mainly influences reproductive function, whereas ERβ is involved in the central nervous system, cardiovascular system, and bone health [54]. Additionally, the expression of pS2 and *ESR1* is regulated by the signaling proteins AKT and ERK via the estrogen receptor pathway. Therefore, in this study, we examined the improvement of female menopause symptoms by evaluating estrogen-like activity and menopause-related biomarkers after treating MCF-7 cells with E2 (positive control) and ASE extract. In this study, treatment with ASE extracts significantly increased the expression of *ESR2* (ERβ mRNA) while decreasing *ESR1* (ERα mRNA), as shown in Fig. 1. Typically, the expression level of ERβ in breast cancer cells is considerably lower than that of ERα; thus, the balance between these two receptors is critical [55]. Numerous studies have suggested that the ERα/ERβ ratio is a key determinant of cellular response, with ERβ often functioning as a negative regulator that inhibits ERα-mediated proliferation [56, 57]. Therefore, the alteration in this ratio observed following ASE treatment suggests that ASE may exert beneficial estrogenic effects while mitigating the proliferative risks associated with ERα overexpression.

To reflect distinct biological mechanisms—specifically, gene transcriptional regulation and the activation of signaling proteins—this study employed a dual exposure time strategy. The changes in the expression of genes such as ERα, ERβ, and pS2 observed in Fig. 1 represent transcriptional regulatory processes involving transcription factor activation and mRNA synthesis; thus, a 48-hour incubation period was applied to sufficiently capture these events. In contrast, the phosphorylation events analyzed in Fig. 2, such as p-AKT and p-ERK, are acute signaling events that occur immediately following estrogen receptor activation [58-62]. To capture these rapid responses at the protein level, we analyzed changes over short time intervals ranging from 0 to 30 min post-treatment. The results confirmed that ASE extracts rapidly activated ER-mediated signaling pathways within minutes of treatment, suggesting that ASE is capable of modulating not only long-term transcriptional regulation but also immediate intracellular signaling cascades.

The ovariectomized (OVX) mouse model is widely used as a representative experimental model for studying postmenopausal osteoporosis caused by estrogen deficiency in women. In this study, when E2 and ASE extract, used as positive controls, were administered to OVX-induced mice for six weeks, changes in body weight, food intake, uterine mRNA expression, tissue weight, cardiovascular indicators, blood ALP and Ca levels, and femur characteristics were observed. It has been reported that OVX-induced estrogen deficiency leads to weight gain. Similarly, in this study, the OVX group exhibited AA and pWAT accumulation, along with increased body weight, compared to the Sham group [63]. Furthermore, E2 and ASE extracts were shown to inhibit weight gain and fat accumulation. These results suggest that ASE extract may help alleviate postmenopausal fat accumulation.

The uterus is regulated by estrogen levels, and ovariectomy causes uterine atrophy. However, studies have reported that estradiol and soy isoflavones alleviate uterine atrophy caused by decreased estrogen levels in ovariectomized mice [64]. In this study, the OVX group showed a decrease in uterine weight compared to the Sham group, and uterine weight tended to increase when E2 and ASE extracts were administered. This increase implies that ASE exerts a mild estrogen-like activity, characteristic of phytoestrogens, which helps alleviate uterine atrophy without inducing excessive tissue proliferation or hypertrophy often observed with potent synthetic estrogens. Additionally, the mRNA expression of *ESR1* in the uterus was higher in the OVX group than in the Sham group. This is because *ESR1* expression is regulated by negative feedback; when estrogen levels are insufficient, this feedback weakens, leading to an increase in *ESR1* mRNA expression [65]. On the other hand, the group administered E2 and ASE extracts showed a decrease in uterine *ESR1* mRNA expression compared to the OVX group. This reduction in *ESR1* expression is due to negative feedback caused by increased estrogen levels. Collectively, these results suggest that ASE acts as a partial estrogen agonist, effectively modulating uterine health through mild estrogenic signaling.

Estrogen plays an important role in skeletal homeostasis, and menopause can lead to osteoporosis [66]. Estrogen intake has been reported to increase bone mineral content and collagen fiber composition while preventing fractures. Additionally, estrogen promotes bone formation by stimulating osteoblast activity and inhibiting osteoclast activity. Through these actions, estrogen helps regulate bone resorption and maintain bone strength. Therefore, in this study, blood ALP and Ca levels were measured to assess changes in bone metabolism. The results showed that blood ALP levels in the OVX group decreased compared to the Sham group, while the E2 and ASE extract administration groups exhibited an increasing trend. Furthermore, micro-CT analysis was performed to evaluate the effect of ASE extract on bone loss. The BMD, BV/TV, and BS/TV values in the OVX group were lower than those in the Sham group. However, in the E2 and ASE extract administration groups, BMD, BV/TV, and BS/TV values increased compared to the OVX group. These findings suggest that ASE extract may help prevent bone loss and reduce the risk of fractures by inhibiting osteoclast activity in postmenopausal women.

Mechanistically, the recovery of bone loss by ASE is closely related to the pathophysiology of postmenopausal osteoporosis. Estrogen deficiency is the primary cause of bone mass reduction in menopausal women, as estrogen signaling—specifically via the PI3K/AKT and MAPK/ERK pathways—plays a critical role in maintaining the balance between osteoblasts (bone formation) and osteoclasts (bone resorption). Since our *in vitro* results (Fig. 2) demonstrated that ASE activates these specific estrogenic signaling cascades, it acts as an effective estrogen substitute. Therefore, ASE likely mitigates OVX-induced osteoporosis by restoring these downregulated signaling pathways in bone tissue, thereby promoting osteoblast survival and inhibiting excessive bone resorption.

Postmenopausal women often experience changes in lipid metabolism and liver function, which are known risk factors for cardiovascular disease (CVD) and other health complications [67, 68]. Therefore, monitoring these metabolic markers provides valuable insights into the systemic effects of estrogen deficiency [69]. In this study, we examined the levels of triglycerides (TG), total cholesterol (TC), HDL-cholesterol (HDL-C), and LDL-cholesterol (LDL-C) to assess metabolic alterations associated with the OVX model.

The results showed that the OVX group exhibited typical dyslipidemic patterns, including decreased HDL-C levels and increased LDL-C, TG, and TC levels compared to the Sham group, consistent with previous reports on estrogen deficiency-induced metabolic deterioration [70]. However, administration of E2 and ASE extracts effectively reversed these trends, increasing HDL-C while reducing LDL-C, TG, and TC levels. It is important to note that while these markers are indicators of cardiovascular health, this study focused on evaluating the ameliorative effects of ASE on menopausal symptoms rather than directly assessing CVD risk outcomes. Therefore, these findings suggest that ASE extract helps restore lipid balance and ameliorate dyslipidemia associated with estrogen deficiency, serving as auxiliary evidence of the extract's beneficial metabolic effects in the postmenopausal state.

Taken together, this study suggests a potential mechanistic link between the intracellular activity of ASE and its physiological benefits in menopausal models. In *in vitro* experiments using MCF-7 cells, ASE demonstrated functionality as a phytoestrogen by upregulating ERβ expression and activating the PI3K/AKT and MAPK/ERK signaling pathways [71]. Notably, phytoestrogens are known to exhibit tissue-selective effects without significantly increasing systemic estrogen levels, owing to their structural similarity to estrogens combined with a higher affinity for ERβ and weaker ERα activity. The finding in this study that ASE increased uterine weight, but to a significantly lesser extent than E2, suggests that ASE may serve as a safe alternative to HRT without the risk of potent uterine proliferative side effects.

Although these molecular responses were observed in breast cancer cell lines, these signaling pathways are widely recognized for their crucial roles in maintaining bone homeostasis and regulating lipid metabolism [72, 73]. In particular, the activation of AKT and ERK signaling is well-documented to promote osteoblast differentiation and survival [74]. Therefore, the significant improvements in bone mineral density (BMD) and lipid profiles observed in the OVX mouse model are consistent with the estrogen-like mechanisms confirmed *in vitro*, suggesting that ASE alleviated menopausal symptoms by modulating these key metabolic signaling pathways. Although this study proposed a mechanism based on mRNA and downstream signaling analyses, direct verification of ERα/ERβ protein expression in future studies is required to more clearly elucidate the estrogen-like mechanism of ASE. Additionally, a limitation of this study is the lack of direct measurement of serum estradiol (E2) levels. While the minimal uterine hypertrophy induced by ASE compared to synthetic E2 suggests that it may not excessively increase systemic estrogen levels, direct verification of serum E2 levels in future research is necessary to clearly elucidate the safety mechanism.

## Conclusion

In this study, the effect of ASE extract on improving menopausal symptoms was investigated using the breast cancer cell line MCF-7 and an ovariectomized (OVX) mouse model through *in vitro* and *in vivo* experiments. The results demonstrated that ASE extract alleviated menopausal symptoms in the OVX model, suggesting its potential as a new alternative for addressing various menopause-related health issues.

## Figures and Tables

**Table 1:** Primer sequences used in this study.

Gene	Direction	Primer sequence (5'-3')
ESR1	Sense	TGGGCTTACTGACCAACCTG
	Anti-sense	CCTGATCATGGAGGGTCAAA
ESR2	Sense	AGAGTCCCTGGTGTGAAGCAA
	Anti-sense	GACAGCGCAGAAGTGAGCATC
TFF1	Sense	CGACGTCCCTCCAGAAGAG
	Anti-sense	CTCTGGGACTAATCACCGTGCTG
hGAPDH	Sense	ACCACCAACTGCTTAGCACC
	Anti-sense	CCATCCACAGTCTTCTGGGT
